# Immunohistochemical localisation of pS2 protein in ductal carcinoma in situ and benign lesions of the breast.

**DOI:** 10.1038/bjc.1993.136

**Published:** 1993-04

**Authors:** Y. A. Luqmani, T. Campbell, S. Soomro, S. Shousha, M. C. Rio, R. C. Coombes

**Affiliations:** Department of Medical Oncology, Charing Cross Hospital Medical School, London, UK.

## Abstract

**Images:**


Br. J. Cancer (1993), 67, 749–753 © Macmillan Press Ltd., 1993

### Immunohistochemical localisation of pS2 protein in ductal carcinoma *in situ* and benign lesions of the breast

Y.A. Luqmani[1], T. Campbell[1], S. Soomro[2], S. Shousha[2], M.C. Rio[3] & R.C. Coombes[1]

*Departments of [1]Medical Oncology, and [2]Histopathology, Charing Cross Hospital Medical School, Fulham Palace Road, London W6 8RF, UK; and [3]INSERM/U184 and CNRS/LGME Institut de Chemie Biologique, Faculte de Medecine, Strasbourg, France.*

**Summary** The expression of pS2 was examined histochemically in paraffin sections taken from biopsy material from patients diagnosed with ductal carcincoma *in situ* (DCIS). Often intense immunoreactivity, to an anti-pS2 monoclonal antibody, was observed in comedo, solid, cribriform and micropapillary types of DCIS, with significant positivity found in 63–67% of cases. In 15 samples analysed, we found a good correlation between pS2 expression and presence of progesterone receptor positive cells, but not with estrogen receptor. There was only a limited degree of correspondence between the cells staining with these anti-sera. Some pS2 positive cells were also seen in normal acini in areas adjacent to cancer but much less frequently in sections of normal breast from reduction mammoplasty. Most normal areas were negative, as were cysts. In benign proliferative conditions (seen in sections with and without DCIS) such as adenosis, sclerosing adenosis, mild and florid ductal epithelial hyperplasia, significant pS2 positivity was seen in about 50% of cases.

These results suggest that there is a progressive increase in pS2 from normal to benign to cancer cells and that this gene is expressed in both the invasive and pre-invasive forms of breast cancer.

The pS2 gene, which was originally isolated by virtue of its estrogen-inducibility (Masiakowski *et al.*, 1982), has been shown to be predominantly associated with estrogen receptor (ER) positive breast cancers (Rio *et al.*, 1987; Skilton *et al.*, 1989; Henry *et al.*, 1989). Limited pS2 immunostaining has also been reported in normal breast and in parts of the ileum (Piggott *et al.*, 1991; Luqmani *et al.*, 1992), as well as more extensive expression in the stomach, but is otherwise absent from the vast majority of normal tissues. It is however, widely expressed in other epithelial cancers (Henry *et al.*, 1991; Luqmani *et al.*, 1989; Rio *et al.*, 1988; Luqmani *et al.*, 1991; Wysocki *et al.*, 1990) as well as in inflammatory conditions of the gastro-intestinal tract (Rio *et al.*, 1991; Seitz *et al.*, 1992). pS2 has significant sequence homology with the (pancreatic) spasmolytic polypeptide (hSP) Wright *et al.*, 1990) with which it is co-expressed in gastric mucosa (Theisinger *et al.*, 1991) and is also secreted into the gastric fluid (Rio *et al.*, 1988).

In a small study we have recently (Shousha *et al.*, manuscript submitted) found pS2 to have no prognostic significance in patients with colo-rectal cancer, but high pS2 levels were predictive of both longer survival (Foekens *et al.*, 1990) and favourable response of breast cancer patients to endocrine therapy (Henry *et al.*, 1989; Skilton *et al.*, 1989) and, in this regard, may be superior to ER (Schwartz *et al.*, 1991). Little is known of the expression of pS2 in early breast cancer. We therefore carried out an immunohistochemical study using archival material obtained from patients who had been diagnosed with ductal carcinoma *in situ* (DCIS), a neoplastic condition in which the malignant cells are confined within the basement membranes of the mammary ducts. This is believed to constitute a fore-runner of the invasive type of carcinoma which involves penetration through the basement membrane into the surrounding stromal tissue.

### Materials and methods

### *Tissues*

Archival paraffin embedded material was used for this study. The tissue blocks containing biopsies taken from patients diagnosed with DCIS, were either from the Histopathology department at Charing Cross Hospital or kindly made available by Mr J.C. Gazet from St George's Hospital. Each case of DCIS was further classified as comedo, solid, cribriform or micropapillary type using widely accepted histological criteria (Page *et al.*, 1987). The blocks containing formalin fixed normal breast tissue from reduction mammoplasty specimens were kindly provided by Dr J. Gomm.

### *Immunohistochemistry*

*pS2* Paraffin sections (5 μm) cut into polylysine coated slides were de-waxed and re-hydrated in phosphate buffered saline (PBS) pH 7.4 after rinsing in alcohol. Endogenous peroxidase was blocked by incubation (15 min at 20°C) in methanol containing 3% hydrogen peroxide. Following a brief rinse in PBS (5 min), sections were incubated with non-immune sheep serum (2.5% in PBS containing 0.5% BSA) to block non-specific Fc receptors. After rinsing in PBS, sections were incubated overnight at 4°C with mouse anti-pS2 monoclonal antibody (diluted 1:150). After washing with more PBS (3 × 5 min), the sections were incubated with biotinylated horse anti-mouse immunoglobulin (1:1000 dilution) (this and subsequent reactions utilised a Vectastain kit) for 30 min. This was washed off by rinsing twice in PBS for 5 min each. The streptavidin-peroxidase complex was added and after 45 min removed by rinsing in PBS (3 × 5 min). Visualisation was achieved by a final incubation in diaminobenzidine (Sigma, UK). After rinsing in tap water, sections were counterstained with Harris haematoxylin. Controls were run in parallel, in which the pS2 antibody was replaced with non-immune serum. The relative degree of staining was assessed as before (Luqmani *et al.*, 1989) and scored from negative ( – ) to highly positive ( + + + ). This method of analysis was preferred to ones which involve estimation of the percentage of cells stained in a number of microscope fields.

### *Estrogen and progesterone receptor*

For ER staining (Elias *et al.*, 1990), 5 μm paraffin sections were kept at 37°C overnight prior to dewaxing. Endogenous peroxidase was blocked by incubation in 3% hydrogen peroxide in methanol. After rinsing with PBS at 37°C, sections were incubated with pronase (Sigma, UK) for 9 min at 37°C followed by washing in ice cold PBS for 3 min and storage at – 20°C for 10 min. After another rinse with PBS at 20°C, blocking reagent (ERICA kit from Abbott Laboratories Ltd UK) was added for 30 min followed by an overnight incubation at 20°C with two drops of the ER

Correspondence: Y.A. Luqmani.
Received 27 May 1992; and in revised form 14 October 1992.

monoclonal antibody. After rinsing in PBS, an additional two drops of ER antibody were added and left for 2 h at 38°C before another PBS rinse and incubation with biotinylated rabbit anti-rat IgG (Vector Laboratories, UK) (1:100 dilution in PBS) for 1 h at 20°C. After washing in PBS, sections were incubated with 0.05% diaminobenzidine (Sigma, UK) and 0.01% hydrogen peroxide containing 0.05% imidazole in Tris-HCl pH 7.2 for 10 min. Sections were then washed in PBS followed by running tap water, briefly immersed in 1% osmium tetroxide, washed in tap water and counterstained with haematoxylin.

For PR (Soomro & Shousha, 1990), paraffin sections prepared as for ER, were covered with normal goat serum for 30 min, then incubated with two drops of PR monoclonal antibody (Abbott Laboratories, UK) at 4°C overnight. After rinsing in 0.2 M Tris buffered saline, sections were incubated with biotinylated anti-rat IgG (1:100 dilution) for 2 h, then rinsed and incubated with avidin-biotin complex (Dako) for 2 h, followed by procedures described for ER, before counterstaining.

In both cases nuclear staining was assessed semi-quantitatively as either negative or positive (weak and strong).

### Results

### *DCIS*

We obtained biopsies from 47 patients originally diagnosed as having DCIS. However, in the sections which we examined, histological evidence of DCIS was seen in only 35 of these. In most cases, there was more than one type of DCIS present on the section, except in the case of the papillary variant, which appeared exclusively, in 9/12 cases. In all but one instance, the occurrence and degree of pS2 positivity was similar for each type when found together. The frequency of significant positivity ( + / + + / + + + ) was the same for all four types, ranging from about 63–67% of cases in which they were observed. There was no consistent difference in the pattern of staining, although in the comedo type, the immunoreactivity was usually associated with the cells on the periphery away from the necrotic center whilst in the other types, it was more uniformly distributed. Figure 1 shows an example of the staining in each type. The staining was generally more intense than we have previously observed with invasive carcinomas. A single case with lobular carinoma *in situ* was weakly positive.

**Table I** pS2 expression in cases with DCIS

| *DCIS type* | *No.* | *pS2 positivity* – | ± | + | + + | + + + |
|---|---|---|---|---|---|---|
| Comedo | 21 | 6(2) | 1 | 6(2) | 2 | 6 |
| Solid | 19 | 3(1) | 4 | 3 | 3 | 6 |
| Cribriform | 11 | 4(1) | 1 | 1 | 1 | 4 |
| Micropapillary | 12 | 2(1) | 2(2) | 5(4) | 1(1) | 2(1) |

Figures in brackets indicate No of cases with one type only in the section: rest contained more than one type.

**Table II** pS2 expression in non-malignant histological conditions

| *Histological feature* | *No.*[a] | *pS2 positivity* – | ± | + | + + |
|---|---|---|---|---|---|
| Normal elements | 33 | 17 | 12 | 3 | 1 |
| Cysts | | 17 | 13 | 3 | 1 |
| Adenosis | 8 | 2 | 2 | 4 | |
| Schlerosing adenosis | 4 | 1 | 1 | 2 | |
| Mild hyperplasia | 3 | | 1 | 2 | |
| Florid ductal hyperplasia | 5 | 2 | 1 | 1 | 1 |

[a]Refers to number of sections in which the particular feature was observed.

**Figure 1** Immunoperoxidase staining of formalin fixed sections showing reactivity with pS2 monoclonal antibody in **a**, solid **b**, comedo **c**, cribriform and **d**, micropapillary DCIS variants. Scale bars represent 70 μM for **a** and 170 μM for **b–d**.

**Figure 2** Parallel formalin fixed sections showing malignant cells in a solid DCIS displaying intense cytoplasmic reactivity with antisera to pS2 **a**, and exclusively nuclear staining with PR antibody **b**, indicating the presence of these two proteins within the same cells. Scale bar represents 120 µM.

*Correlation of pS2 with ER and PR*

In order to determine whether pS2 was present in exactly the same cells as PR and whether it was restricted to those that were also ER positive, we examined 12 cases containing DCIS that were pS2 positive and three that were negative, for the presence of ER and PR, by staining parallel sections. We found that 10/12 of the pS2 positive samples had PR positive cells but only 5/12 had ER positive staining. All three of the pS2 negative samples were PR negative. There was some degree of correspondence between the cells staining with the pS2 and PR anti-sera, but this was not always the case; sometimes different tumour cells in the same section stained with only one or other of the anti-sera. Thus although there was a good overall correlation between pS2 and PR, this did not necessarily reflect co-expression within the same cells. Figure 2 shows parallel sections containing a focus of solid DCIS in which the cells did express both pS2 and PR, but were negative for ER.

*Non-malignant elements*

In many samples (both those containing DCIS and those in which no cancer cells were observed) we also found normal as well as other non-malignant elements. The staining pattern observed with these is summarised in Table II. Normal lobules were found in 33 of the 46 cases and significant pS2 staining was seen in four of these, although some positivity was also seen in 12 other cases, predominantly as an intense reaction in a few scattered acini. The majority of cases with adenosis, sclerosing adenosis and either mild or florid ductal epithelial hyperplasia, had weak (mostly ± / + compared to DCIS) immunostaining in those areas. An example of one of these is shown in Figure 3a: panel b shows a rare case of staining in cells within a lobule of normal appearance whilst panel c shows a similar lobule in which most of the normal acini are negative, but a few cells, which appear to have atypical nuclei, as positive. In contrast, positive staining in cysts was seen in only 4/17 cases in which they were found. Although the number of cases examined was relatively small, we observed no obvious difference in the staining of the hyperplastic conditions whether or not the section also had elements of DCIS.

*Reduction mammoplasty specimens*

For comparison, we stained sections of specimens obtained from breast reduction surgery, which apart from one case of virginal hyperplasia, had cytologically normal ducts and lobules. In three cases there was no staining at all while in the other three, including the case mentioned above, a few epithelial cells showed weak ( ± ) reactivity.

### Discussion

In this study we have shown that pS2, hitherto examined predominantly in invasive breast cancers (Rio *et al.*, 1987; Henry *et al.*, 1989; Skilton *et al.*, 1989), is sporadically expressed in apparently normal epithelia, especially in areas adjacent to cancer, but much less so in normal breast alveoli found in reduction mammoplasty specimens where there are no malignant cells. Piggott *et al.*; (Piggott *et al.*, 1991) also observed positive staining in normal breast but in a much higher proportion of samples, while Rio *et al.*; (Rio *et al.*, 1987) reported no staining of normal cells at the periphery of cancers. However, in specifically looking for this difference, we have found that there is a progressive increase in immunoreactivity, from normal to benign hyperplastic

**Figure 3** pS2 immunostaining of areas of non-malignant breast within sections containing DCIS. **a**, shows reactivity in cells within an apparently normal breast lobule and **b**, in an area of sclerosing adenosis. **c**, shows an example of a normal breast lobule in which one acini has abnormal looking cells which are strongly positive. Scale bar represents 400 µM in **a** and **b** and 40 µM in **c**.

lesions, to neoplastic cells, including those that have not yet become invasive. As seen in Figure 3c, whereas most normal cells are pS2 negative, immunoreactivity is associated with the appearance of cells displaying abnormal characteristics.

Cysts, which may be likened to dilated acini with little other signs of abnormality, were a commonly observed feature and only a few expressed pS2 protein. Sclerosing adenosis, a relatively common benign lesion of the mammary lobule, whose presence is thought to be associated with an increased risk for developing breast carcinoma (Jensen *et al.*, 1989) and in which DCIS has been reported to develop occasionally (Eusebi *et al.*, 1989) was in contrast, predominantly pS2 positive. Thus it is still unclear whether the pS2 protein has any real physiological role in the normal breast; it's appearance there may simply reflect beginnings of abnormalities restricted to sporadic cells. Increased incidence of pS2 expression in epithelial hyperplasia is interesting in view of recent opinions regarding this as one of the most critical potential precursors of breast cancer with an increased risk of 4–7 fold (Bulbrook & Miller, 1980).

The detection of DCIS, regarded as an obligate precursor of invasive cancer (Carter & Eggleston, 1977), appears to be increasing, presumably as a direct result of more widespread mammographic screening. This presents an opportunity for identifying genetic changes involved in tumour invasion. DCIS shows a marked expression of pS2, characterised by often intense focal staining which, in our past experience with this antibody, is not a general feature of invasive cancers in which the staining is less intense and more heterogeneous (Luqmani *et al.*, 1992). A similar finding has been reported for c-erbB-2 staining of comedo carcinomas where these have been seen with invasive elements (Maguire *et al.*, 1991). Our results would suggest that pS2 is already highly expressed in malignant cells before they have acquired the propensity for invasion, as indeed is c-erbB-2 (Maguire *et al.*, 1991). However, unlike the latter (Bartkova *et al.*, 1990), we found pS2 to be present in both the comedo and non-comedo types. Interestingly, the frequency of ER staining of paraffin embedded DCIS cases has been reported (Giri *et al.*, 1989) to be much greater in cribriform, papillary and solid types (>50%) as compared with comedo (<20%). This discordance between pS2 and ER staining, also reflected by our own ER results, suggests that pS2 expression may not be obligatorily linked to that of ER as originally envisaged (Rio *et al.*, 1987). Even the close correlation of pS2 with PR was due as often as not, to an assessment of the overall staining on parallel sections, than to co-expression in the same cells.

Although the comedo type of DCIS appears to have a histologically more aggressive phenotype and seems to be associated with a greater risk of advancement to invasive cancer, there is considerable uncertainty regarding diagnostic criteria for these lesions and a lack of reliable prognostic markers to predict which tumours are likely to become invasive. As mastectomy has until recently been the standard treatment, information on disease recurrence is sparse, but several small studies (Campbell *et al.*, 1992; Fuqua *et al.*, 1991) have indicated subsequent development of invasive cancer only in a minority of patients with DCIS. The urgent need for prognostic markers, to provide less radical breast conserving treatment, is self evident and is likely to become a much debated issue (Fentiman, 1990) as the number of screen detected early cancers rise. In view of our initial observations we consider that it would be of value to examine pS2 expression in a larger series where follow-up data is available.

We are grateful to P. Chambon for his continued support in supplying the pS2 antisera, and to the Cancer Research Campaign for financial support in the form of a Programme grant to RCC and YAL.

### References

BARTKOVA, J., BARNES, D.M. MILLIS, R.R. & GULLICK, W.J. (1990). Immunohistochemical demonstration of c-erbB-2 protein in mammary ductal carincoma *in situ*. *Human Pathol.*, **21**, 1164–1167.

BULBROOK, R.D. & MILLER, A.B. (1980). Special Report. The epidemiology and etiology of breast cancer. *N. Engl. J. Med.*, **303**, 1246–1248.

CAMPBELL, T., SKILTON, R.A., COOMBES, R.C., SHOUSHA, S., GRAHAM, M.D. & LUQMANI, Y.A. (1992). Isolation of a lactoferrin cDNA clone and its expression in human breast. *Br. J. Cancer*, **65**, 19–26.

CARTER, D. & EGGLESTON, J.C. The pathology of breast cancer. In *Current Trends in the Management of Breast Cancer*, edited by Baker, R.R. London: Bailliere Tindall, 1977, p. 21–65.

ELIAS, J.M., HEIMANN, A., CAIN, T., MARGIOTTA, M., GALLERY, F. & GOMES, C. (1990). Estrogen receptor localisation in paraffin sections by enzymatic digestion, repeated applications of primary antibody, and imadazole. *J. Histotechnol.*, **13**, 29–33.

EUSEBI, V., GUIDO, M.D. & BUSSOLATI, M.D. (1989). Carcinoma in situ in sclerosing adenosis of the breast: an immunocytochemical study. *Seminars in Diag. Pathol.*, **2**, 146–152.

FENTIMAN, I.S. (1990). Treatment of screen detected ductal carcinoma in situ: a silver lining within a grey cloud? *Br. J. Cancer*, **61**, 795–796.

FOEKENS, J.A., RIO, M.C., SEGUIN, P., VAN PUTTEN, W.L.J., FAUQUE, J., NAP, M., KLIJN, J.G.M. & CHAMBON, P. (1990). Prediction of relapse and survival in breast cancer patients by pS2 protein status. *Cancer Res.*, **50**, 2832–2837.

FUQUA, S.A.W., FITZGERALD, S.D., CHAMNESS, G.C., TANDON, A.K., MCDONNEL, D.P., NAWAZ, Z., O'MALLEY, B.W. & MCGUIRE, W.L. (1991). Variant human breast tumor estrogen receptor with constitutive transcriptional activity. *Cancer Res.*, **51**, 105–109.

GIRI, D.D., DUNDAS, S.A.C., NOTTINGHAM, J.F. & UNDERWOOD, J.C.E. (1989). Oestrogen receptors in benign epithelial lesions and intraduct carcinomas of the breast: an immunohistological study. *Histopathology*, **15**, 575–584.

HENRY, J.A., NICHOLSON, S., HENNESSY, C., LENNARD, T.W.J., MAY, F.E.B. & WESTLEY, B.R. (1989). Expression of the estrogen regulated pNR-2 mRNA in human breast cancer: relation to estrogen receptor mRNA levels and response to tamoxifen therapy. *Br. J. Cancer*, **61**, 32–38.

HENRY, J.A., BENNETT, M.K., LEVETT, D., MAY, F.E.B. & WESTLEY, B.R. (1991). Expression of the pNR/pS2 protein in diverse human epithelial tumours. *Br. J. Cancer*, **64**, 677–683.

JENSEN, R.A., PAGE, D.L. DUPONT, W.D. & ROGERS, L.W. (1989). Invasive breast cancer risk in women with sclerosing adenosis. *Cancer*, **64**, 1977–1983.

LUQMANI, Y.A., BENNETT, C., PATERSON, I., CORBISHLEY, C.M., RIO, M.C., CHAMBON, P. & RYALL, G. (1989). Expression of the pS2 gene in normal, benign and neoplastic human stomach. *Int. J. Cancer*, **44**, 806–812.

LUQMANI, Y.A., RYALL, G., SHOUSHA, S. & COOMBES, R.C. (1992). An immunohistochemical survey of pS2 expression in epithelial cancers. *Int. J. Cancer*, **50**, 302–304.

MAGUIRE, H.C., GREENE, M.I. & YEH, I.T. (1991). The c-erbB-2 oncogene is expressed similarly in in situ and in adjacent invasive ductal adenocarcinomas of the female breast. *Proc. Am. Assoc. Cancer Res.*, **32**, 293. (Abstract).

MASIAKOWSKI, P., BREATHNACH, R., BLOCH, J., GANNON, K., KRUST, A. & CHAMBON, P. (1982). Cloning of cDNA sequences of hormone regulated genes from MCF-7 human breast cancer cell line. *Nucleic Acids. Res.*, **10**, 7895–7903.

PAGE, D.L., ANDERSON, L. & ROGERS, L.W. (1987). Carcinoma *in situ*. In *Diagnostic Histopathology of the Breast*, Churchill Livingstone, p. 157.

PIGGOTT, N.H., HENRY, J.A., MAY, F.E.B. & WESTLEY, B.R. (1991). Antipeptide antibodies against the pNR-2 oestrogen regulated protein of human breast cancer cells and detection of pNR-2 expression in normal tissues by immunohistochemistry. *J. Pathol.*, **163**, 95–104.

RIO, M.C., BELLOCQ, J.P., GAIRARD, B., RASMUSSEN, U.B., KRUST, A., KOEHL, C., CALDEROLI, C., SCHIFF, V., RENAUD, R. & CHAMBON, P. (1987). Specific expression of the pS2 gene in sub-classes of breast cancers in comparison with expression of the estrogen and progesterone receptors and the oncogene erbB2. *Proc. Natl Acad. Sci.*, **84**, 9243–9247.

RIO, M.C., BELLOCQ, J.P., DANIEL, J.Y., TOMASETTO, C., LATHE, R., CHENARD, M.P., BATZENSCHLAGER, A. & CHAMBON, P. (1988). Breast cancer-associated protein: synthesis and secretion by normal stomach mucosa. *Science*, **241**, 705–708.

RIO, M.C., CHENARD, M.P., WOLF, C. & CHAMBON, P. (1991). Induction of pS2 and hSP genes as markers of mucosal ulceration of the digestive tract. *Gastroenterology*, **100**, 375–379.

SCHWARTZ, L.H., KOERNER, F.C., EDGERTON, S.M., SAWICKA, J.M., RIO, M.C., BELLOCQ, J.P., CHAMBON, P. & THOR, A.D. (1991). pS2 expression and response to hormonal therapy in patients with advanced breast cancer. *Cancer Res.*, **51**, 624–628.

SEITZ, G., THELSINGER, B., TOMASETTO, G., RIO, M.C., CHAMBON, P., BLIN, N. & WELTER, G. (1991). Breast cancer associated protein pS2 expression in tumors of the biliary tract. *Am. J. Gastroenterol.*, **86**, 1491–1494.

SKILTON, R.A., LUQMANI, Y.A., MCCLELLAND, R.A. & COOMBES, R.C. (1989). Characterisation of a messenger RNA selectively expressed in human breast cancer. *Br. J. Cancer*, **60**, 168–175.

SOOMRO, S. & SHOUSHA, S. (1990). Demonstration of progesterone receptors in paraffin wax sections of breast carcinoma. *J. Clin. Pathol.*, **43**, 671–674.

THEISINGER, B., WELTER, C., SEITZ, G., RIO, M.C., LATHE, P., CHAMBON, P. & BLIN, N. (1991). Expression of the breast cancer associated gene pS2 and the pancreatic spasmolytic polypeptide gene (hSP) in diffuse type of stomach carcinoma. *Eur. J. Cancer*, **27**, 770–773.

WRIGHT, N.A., POULSOM, R., STAMP, G.W., HALL, P.A., JEFFREY, R.E., LONGCROFT, J.M. RIO, M.C., TOMASETTO, C. & CHAMBON, P. (1990). Epidermal growth factor (EGF/URO) induces expression of regulatory peptides in damages human gastrointestinal tissues. *J. Pathol.*, **162**, 279–284.

WYSOCKI, S.J., HAHNEL, E., MASTERS, A., SMITH, V., MCCARTNEY, A.J. & HAHNEL, R. (1990). Detection of pS2 messenger RNA in gynaecological cancers. *Cancer Res.*, **50**, 1800–1802.


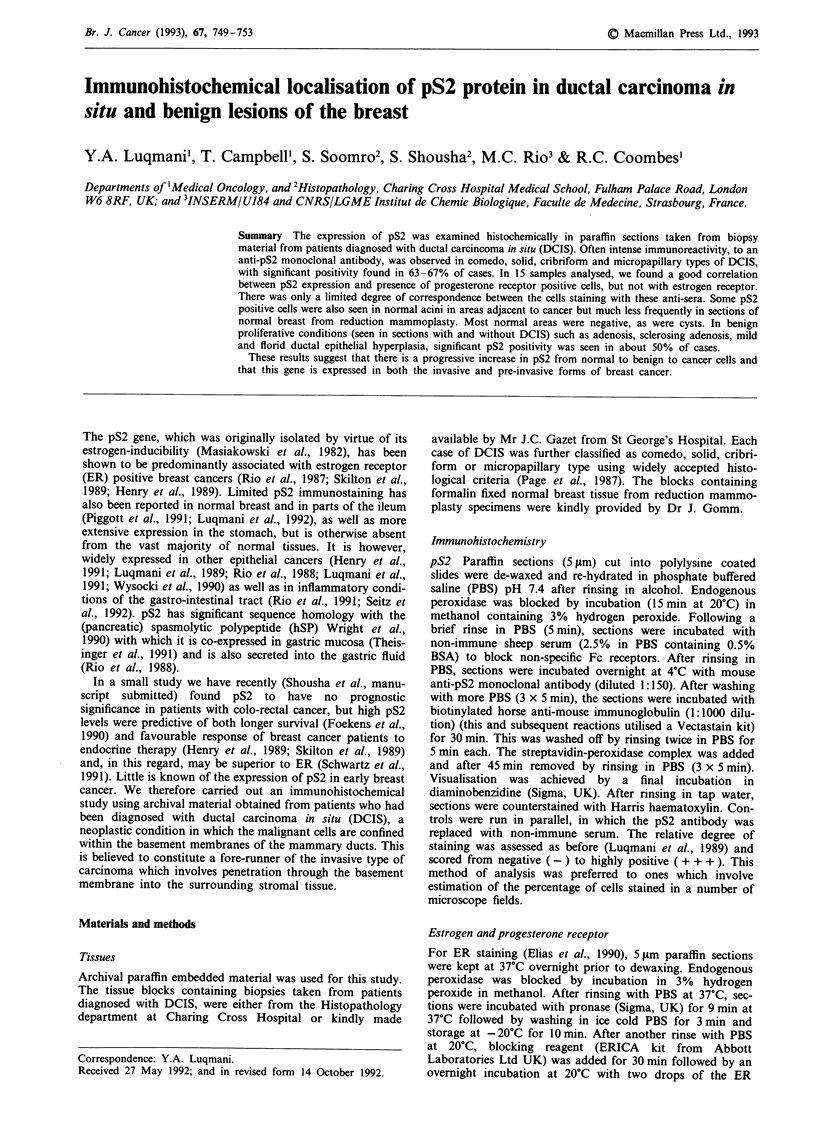

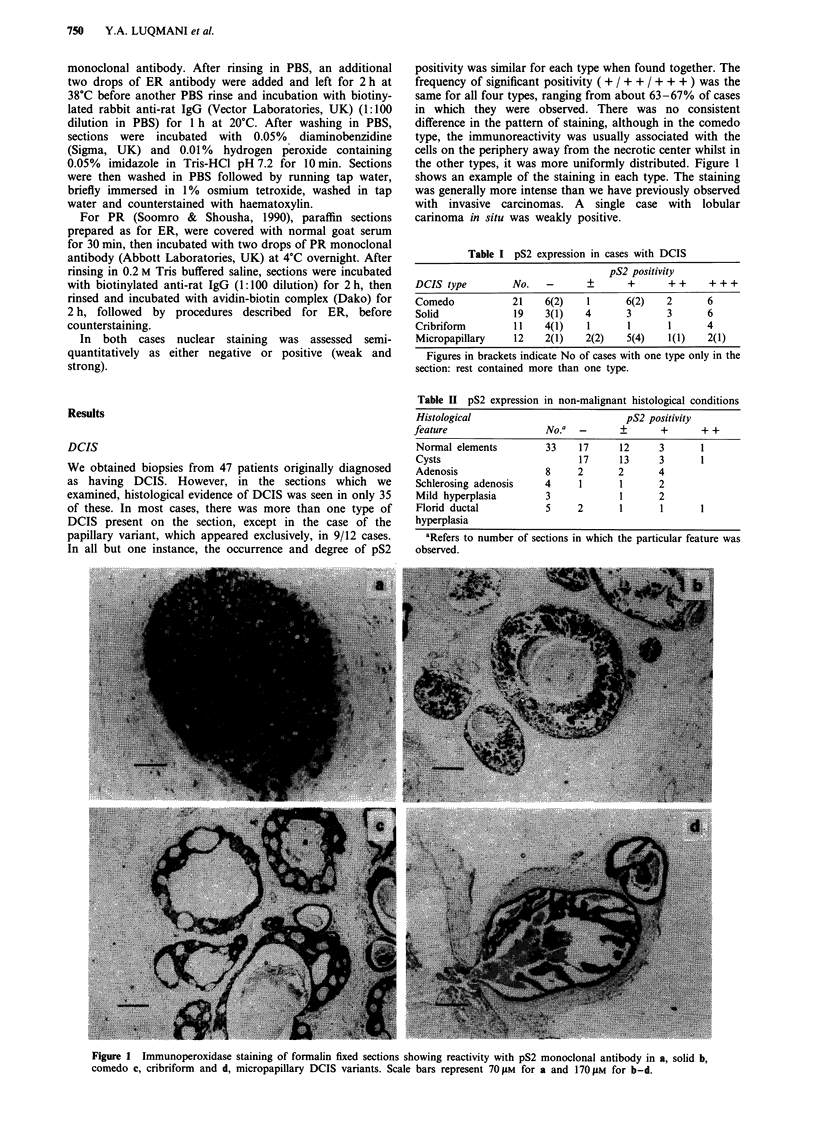

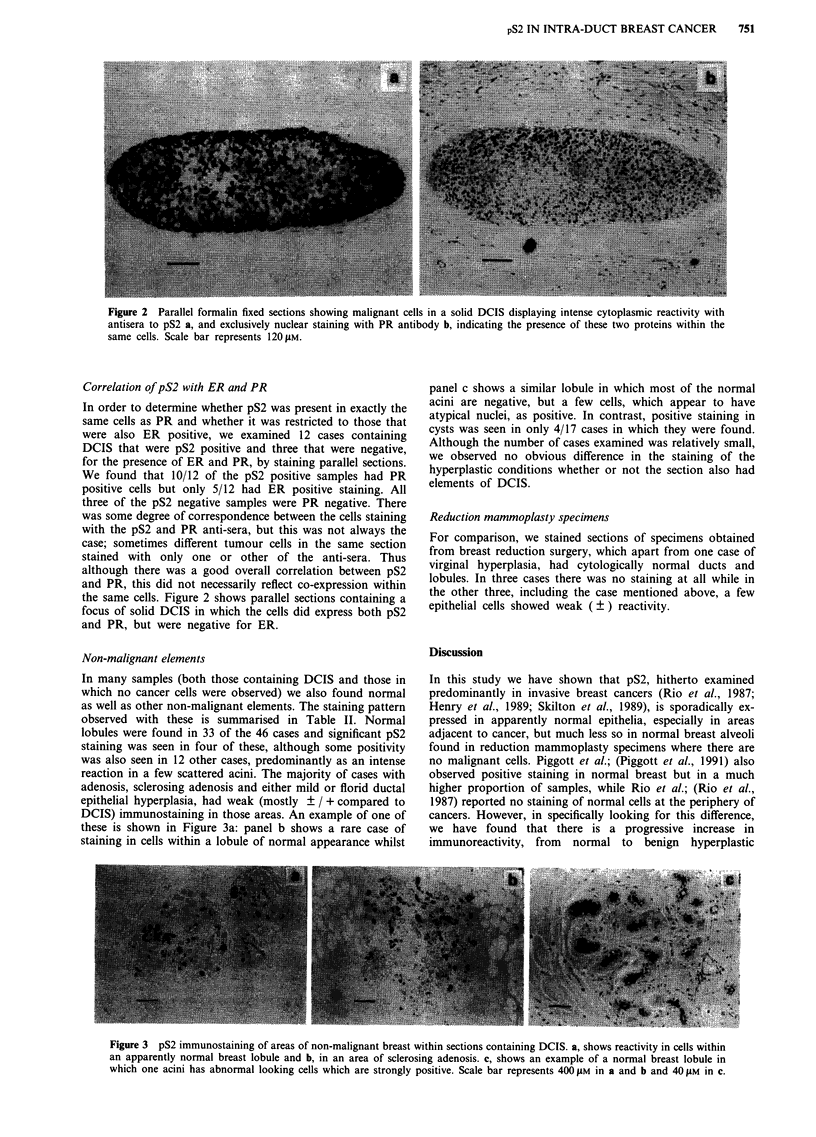

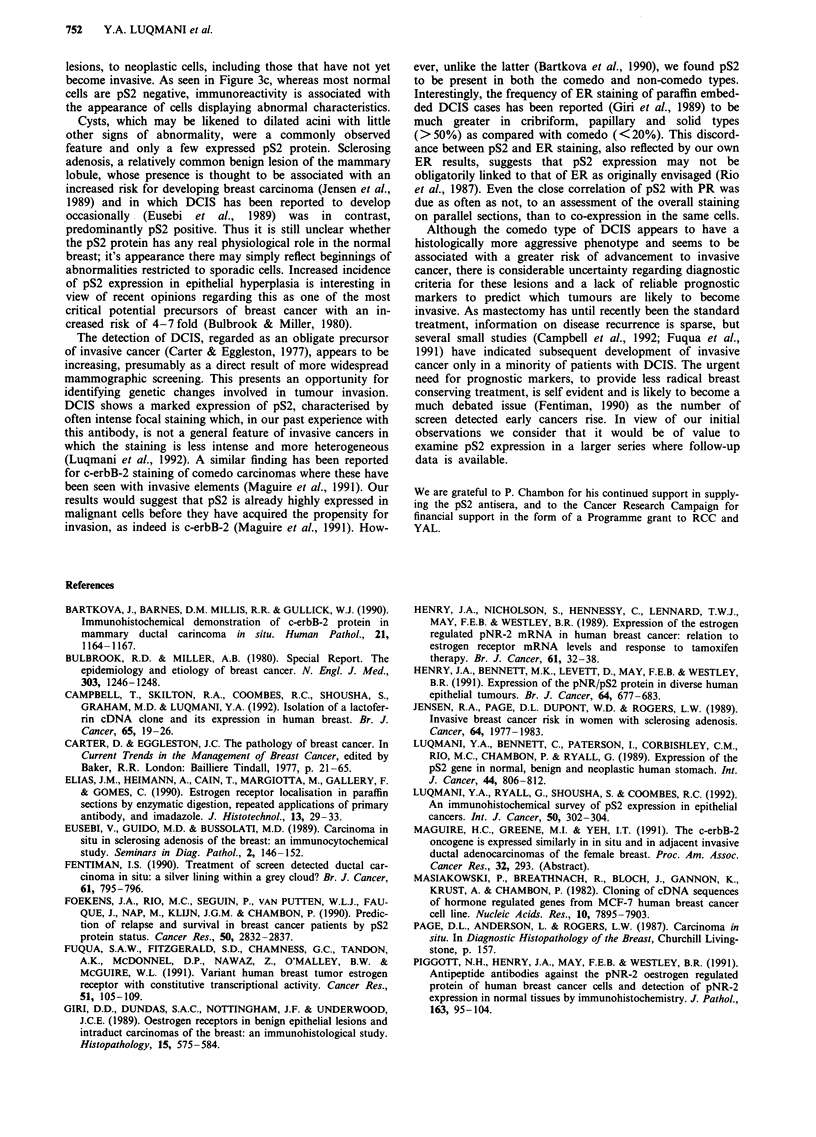

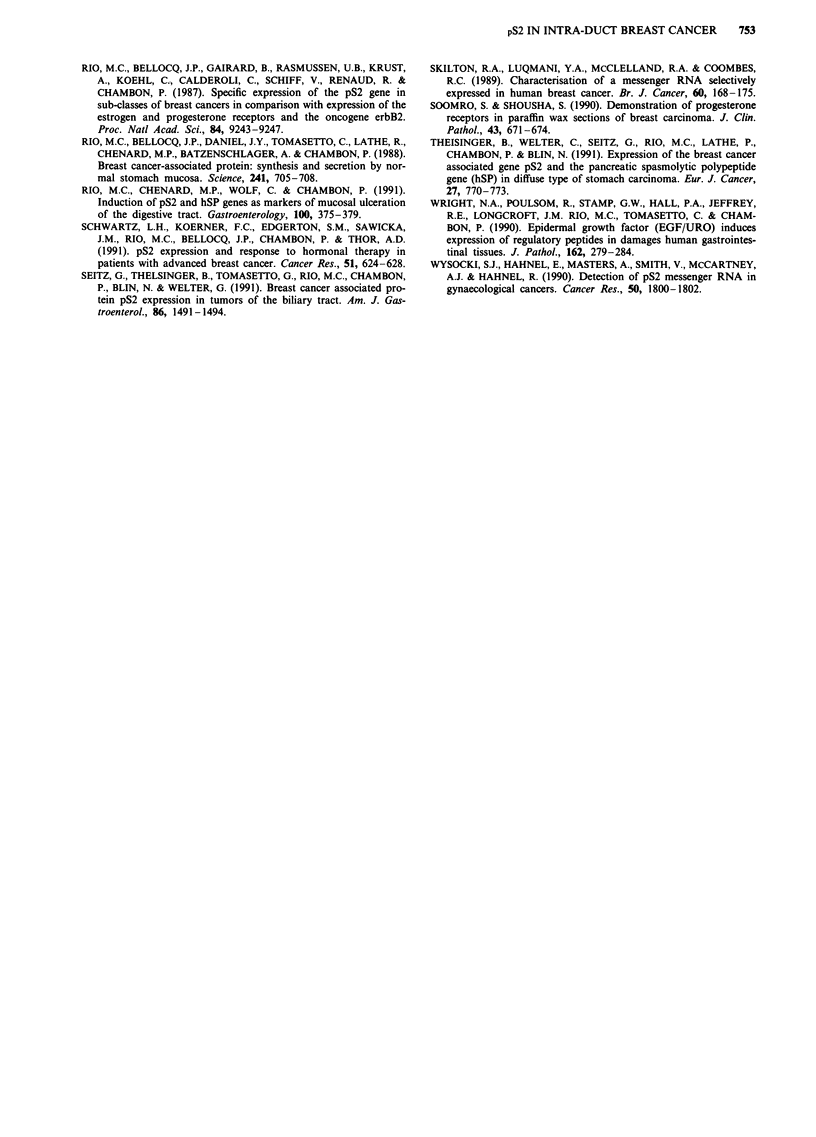


## References

[OCR_00383] Bartkova J., Barnes D. M., Millis R. R., Gullick W. J. (1990). Immunohistochemical demonstration of c-erbB-2 protein in mammary ductal carcinoma in situ.. Hum Pathol.

[OCR_00394] Campbell T., Skilton R. A., Coombes R. C., Shousha S., Graham M. D., Luqmani Y. A. (1992). Isolation of a lactoferrin cDNA clone and its expression in human breast cancer.. Br J Cancer.

[OCR_00411] Eusebi V., Collina G., Bussolati G. (1989). Carcinoma in situ in sclerosing adenosis of the breast: an immunocytochemical study.. Semin Diagn Pathol.

[OCR_00416] Fentiman I. S. (1990). Treatment of screen detected ductal carcinoma in situ: a silver lining within a grey cloud?. Br J Cancer.

[OCR_00427] Fuqua S. A., Fitzgerald S. D., Chamness G. C., Tandon A. K., McDonnell D. P., Nawaz Z., O'Malley B. W., McGuire W. L. (1991). Variant human breast tumor estrogen receptor with constitutive transcriptional activity.. Cancer Res.

[OCR_00434] Giri D. D., Dundas S. A., Nottingham J. F., Underwood J. C. (1989). Oestrogen receptors in benign epithelial lesions and intraduct carcinomas of the breast: an immunohistological study.. Histopathology.

[OCR_00447] Henry J. A., Bennett M. K., Piggott N. H., Levett D. L., May F. E., Westley B. R. (1991). Expression of the pNR-2/pS2 protein in diverse human epithelial tumours.. Br J Cancer.

[OCR_00440] Henry J. A., Nicholson S., Hennessy C., Lennard T. W., May F. E., Westley B. R. (1990). Expression of the oestrogen regulated pNR-2 mRNA in human breast cancer: relation to oestrogen receptor mRNA levels and response to tamoxifen therapy.. Br J Cancer.

[OCR_00452] Jensen R. A., Page D. L., Dupont W. D., Rogers L. W. (1989). Invasive breast cancer risk in women with sclerosing adenosis.. Cancer.

[OCR_00463] Luqmani Y. A., Ryall G., Shousha S., Coombes R. C. (1992). An immunohistochemical survey of pS2 expression in human epithelial cancers.. Int J Cancer.

[OCR_00457] Luqmani Y., Bennett C., Paterson I., Corbishley C. M., Rio M. C., Chambon P., Ryall G. (1989). Expression of the pS2 gene in normal, benign and neoplastic human stomach.. Int J Cancer.

[OCR_00474] Masiakowski P., Breathnach R., Bloch J., Gannon F., Krust A., Chambon P. (1982). Cloning of cDNA sequences of hormone-regulated genes from the MCF-7 human breast cancer cell line.. Nucleic Acids Res.

[OCR_00389] Miller A. B., Bulbrook R. D. (1980). The epidemiology and etiology of breast cancer.. N Engl J Med.

[OCR_00485] Piggott N. H., Henry J. A., May F. E., Westley B. R. (1991). Antipeptide antibodies against the pNR-2 oestrogen-regulated protein of human breast cancer cells and detection of pNR-2 expression in normal tissues by immunohistochemistry.. J Pathol.

[OCR_00502] Rio M. C., Bellocq J. P., Daniel J. Y., Tomasetto C., Lathe R., Chenard M. P., Batzenschlager A., Chambon P. (1988). Breast cancer-associated pS2 protein: synthesis and secretion by normal stomach mucosa.. Science.

[OCR_00497] Rio M. C., Bellocq J. P., Gairard B., Rasmussen U. B., Krust A., Koehl C., Calderoli H., Schiff V., Renaud R., Chambon P. (1987). Specific expression of the pS2 gene in subclasses of breast cancers in comparison with expression of the estrogen and progesterone receptors and the oncogene ERBB2.. Proc Natl Acad Sci U S A.

[OCR_00508] Rio M. C., Chenard M. P., Wolf C., Marcellin L., Tomasetto C., Lathe R., Bellocq J. P., Chambon P. (1991). Induction of pS2 and hSP genes as markers of mucosal ulceration of the digestive tract.. Gastroenterology.

[OCR_00423] Rubin A. L. (1990). Suppression of transformation by and growth adaptation to low concentrations of glutamine in NIH-3T3 cells.. Cancer Res.

[OCR_00513] Schwartz L. H., Koerner F. C., Edgerton S. M., Sawicka J. M., Rio M. C., Bellocq J. P., Chambon P., Thor A. D. (1991). pS2 expression and response to hormonal therapy in patients with advanced breast cancer.. Cancer Res.

[OCR_00518] Seitz G., Thelsinger B., Tomasetto G., Rio M. C., Chambon P., Blin N., Welter G. (1991). Breast cancer-associated protein pS2 expression in tumors of the biliary tract.. Am J Gastroenterol.

[OCR_00524] Skilton R. A., Luqmani Y. A., McClelland R. A., Coombes R. C. (1989). Characterisation of a messenger RNA selectively expressed in human breast cancer.. Br J Cancer.

[OCR_00528] Soomro S., Shousha S. (1990). Demonstration of progesterone receptors in paraffin wax sections of breast carcinoma.. J Clin Pathol.

[OCR_00533] Theisinger B., Welter C., Seitz G., Rio M. C., Lathe R., Chambon P., Blin N. (1991). Expression of the breast cancer associated gene pS2 and the pancreatic spasmolytic polypeptide gene (hSP) in diffuse type of stomach carcinoma.. Eur J Cancer.

[OCR_00543] Wright N. A., Poulsom R., Stamp G. W., Hall P. A., Jeffery R. E., Longcroft J. M., Rio M. C., Tomasetto C., Chambon P. (1990). Epidermal growth factor (EGF/URO) induces expression of regulatory peptides in damaged human gastrointestinal tissues.. J Pathol.

[OCR_00547] Wysocki S. J., Hahnel E., Masters A., Smith V., McCartney A. J., Hahnel R. (1990). Detection of pS2 messenger RNA in gynecological cancers.. Cancer Res.

